# “Fantasy Points” associated with Professional Athlete Performance after Lumbar Discectomy or Microdiscectomy

**DOI:** 10.51894/001c.30766

**Published:** 2022-02-24

**Authors:** Marvin Kajy, Devan O. Higginbotham, Guy Ball, Rahul Vaidya

**Affiliations:** 1 Cardiovascular Disease Spectrum Health; 2 Orthopaedic Surgery Detroit Medical Center; 3 Orthopaedic Surgery McLaren Oakland; 4 Orthopaedic Trauma Detroit Receiving Hospital

**Keywords:** athlete, discectomy, spine, performance, surgery, microdiscectomy, lumbar, professional athlete, recovery

## Abstract

**INTRODUCTION:**

The treatment of Lumbar Disc Herniation (LDH) in elite athletes is a debated topic that lacks consensus in the literature due to varying outcome reporting methods. The objective of this study was to quantify the overall performance of a sample of professional athletes before and after receiving a lumbar discectomy or microdiscectomy in a cohort of players in the National Football League (NFL), National Basketball Association (NBA), National Hockey League (NHL) and Major League Baseball (MLB).

**METHODS:**

The authors identified publicly accessible data from a cohort of different types of professional players who received either a lumbar discectomy or a microdiscectomy. These records were identified through newspaper archives, injury reports, player profiles and press releases between 1993 through 2015. Fantasy and Wins Above Replacement (WAR) scores were calculated for each player.

**RESULTS:**

A total of 38 professional players met study inclusion criteria. NFL players had the lowest return-to-play (RTP) at nine of 14 (64%). The RTP for NBA, NHL and MLB players were comparable with 6/7 (86%) vs 8/9 (89%) vs 7/8 (88%). NFL players had the lowest average career length after surgery at 34.8 months, while NBA players had the longest average career length at 48 months. MLB players on average required the longest time to return to presurgical level of performance (24 months) and required the longest average recovery time at 12 months.

**CONCLUSIONS:**

Based on these results, the average performance of most elite athletes are likely to decrease after undergoing a lumbar discectomy. Although it appears that performance peaks in the initial years after the operation for some players, there was an overall long-term decline in this sample of elite athletes. Study limitations included small sample size, lack of controlling for possible confounding variables (e.g., age, etc.) and use of variable reporting sources. Additional studies with larger sample sizes and age-matched controls are needed to examine the effects of lumbar discectomy more comprehensively in elite athletes.

## INTRODUCTION

Symptomatic lumbar disc herniation (LDH) is common in the general population.^1,2^ The majority of disc herniations occur between L4-L5 levels and L5-S1 levels.^3,4^ The clinical manifestation of a LDH can vary from completely asymptomatic to excruciating low back pain, sciatica weakness and sensory loss.^5^ Surgical management of LDH is commonly performed via a discectomy (i.e., removal of herniated disc materials) or a microdiscectomy (i.e., use of a specialized microscope to allow a larger view of the herniated disc through use of a smaller incision).^5^

The success of such an operation is generally evaluated by relief of symptoms and the ability of the patient to return to his/her daily functioning.^5^ Although this expectation may hold true for the general population, this may not apply to professional athletes who subject their bodies to greater stress and demands. Professional athletes are predisposed to LDH, especially those playing in contact sports.^6,7^ It has been demonstrated that a discectomy for carefully selected patients with sciatica due to LDH can provide faster relief from an acute attack than conservative management, although longer-term effects on the natural history of the underlying disc disease remain unclear.^8^

In 2011, Hsu et al. investigated outcomes after the diagnosis of LDH in a sample of professional athletes of American football, baseball, hockey, and basketball.^9^ This group reported that after the diagnosis of LDH, 280 of 342 (82%) professional athletes successfully returned to play with an average subsequent career length of 3.4 years. In addition, 184 of 226 (81%) of players in the study who received a discectomy successfully returned to play, on average, for 3.3 years after surgery.^9^ Other studies have reported that professional players returned to play at an average ranging from 10.8 to 34.8 weeks after undergoing a lumbar microdiscectomy.^7,10,11^

The clinical outcomes of LDH after a discectomy have been well studied in the general population. However, the treatment of LDH in elite athletes is a debated topic that lacks consensus in the literature.^12^ Elite athletes may experience unnatural forces on their spines which may differ from the general population and these athletes are concerned about different parameters after treatment that are unique to their careers. These patient outcomes have been generally measured using non-report measures such as return-to-play (RTP) rates, career length, and performance-based outcomes after surgical treatment.^9,13^

Fantasy sports games have been used to provide real-life information about a player’s performance known as “fantasy scores”. Such “fantasy scores” have been observed as a validated form of performance in professional athletes after injury.^14^ “Fantasy scores” are a compilation of multiple statistics to provide a score to measure in-game production of individual National Football League (NFL), National Basketball Association (NBA) and National Hockey League (NHL) players. Wins Above Replacement (WAR) is also commonly used form of fantasy scoring in Major League Baseball (MLB) as it measures a player’s value in all facets of the game by deciphering how many more wins an athlete is worth than a replacement-level player at the same position.^15^

### Objective of Study

The aim of this study was to quantify the performance of professional athletes before and after a lumbar discectomy or a microdiscectomy using assigned fantasy scores in a cohort of NFL, NBA, NHL and MLB players. Prior studies have assessed clinical success based on the return to play rate (RTP), recovery time, and career length.^7,9–12,16–18^ The objective of this study was to be the first to assess operative treatment of different types of elite athletes based on player performance outcomes utilizing their fantasy scores and wins above replacement (WAR).

In addition, the authors examined the RTP, average career length, average length of time to return to presurgical performance and recovery time. The authors’ primary hypothesis was that elite athletes would exhibit a decrease in performance after undergoing a lumbar discectomy or a microdiscectomy because of the unusual physical demands.

## METHODS

Using a previously published methodology,^17–20^ sample players who underwent a lumbar discectomy or a microdiscectomy were identified through publicly accessible newspaper archives, injury reports, player profiles and press releases between 1993-2015. Main sources of information came from ESPN (http://espn.go.com), NFL (http://www.nba.com), NBA (http://www.nba.com), NHL (http://www.nhl.com) and MLB (http://www.mlb.com).

Inclusion criteria included professional athletes who missed at least one game during their regular or postseason season after receiving a lumbar discectomy operation. Player information including primary position, dates of operations, number of games played, fantasy scores and WAR were compiled. For NFL, NBA and NHL players, this information was obtained from ROTOWORLD (http://www.rotoworld.com). Player information for MLB players were obtained from FANGRAPHS (http://www.fangraphs.com). Both data sources contained in-depth sports reporting and sponsor fantasy leagues on their respective platforms.

When analyzing a professional athlete’s performance, it is advised that one should use more than one metric at a time.^14^ The analyses for this study utilized fantasy points and WAR to track a player’s performance from season to season. Fantasy points and WAR were calculated by the authors based on multiple per game performance measures. beginning at zero and could be negative or positive depending upon their respective performance. This method assigned each player a “value” based on his success during a given season and permitted the authors to track the trajectory of sample players as they progressed through their professional career.

Again, fantasy scores for NFL, NBA and NHL players were obtained from ROTOWORLD. To generate fantasy points for sample players, the ROTOWORLD editors generated rankings based on how they projected the player would likely perform after surgery. These ranking projections were primarily based on fantasy point calculations and other factors such as injury risks, potential to grow, reliability, trade prospects, ease of replacement in other categories, and possible professional team and/or organizational issues which may affect their future player performance evaluations.^21,22^

The fantasy scores for NFL players were calculated from a sum. To illustrate, the action that a player performs was worth a specific point. At the end of the season, the sum yielded a fantasy score for the player. Standard points for each action for non-defensive players and defensive players can be seen in Tables 1 and 2, respectively.

**Table 1.** Outline of how the fantasy points for NFL non-defensive players are calculated

**Nondefense Players**	
**Actions**	**Value**
All touchdowns	6 points
Passing yards	1 point/20 yards
Rushing yards	1 point/ 10 yards
Receiving yards	1 point/ 10 yards
Interception	-2 points
Fumble lost	-1 point
Field goal under 39 yards	3 points
Field goal 40-49 yards	4 points
Field goal 50+ yards	5 points
Points after touchdown/ extra point	1 point

**Table 2.** Outline of how the fantasy points for NFL defensive players are calculated

**Defensive Players**	
**Actions**	**Value**
Safety	2 points
Sack	2 points
Interception	2 points
Fumble recovery	2 points
Defensive touchdowns	6 points

Next, the fantasy points in the NHL are calculated using formulas based on a player’s position. Specifically, the formula for defensemen was:

$$\begin{array}{rl} FantasyScore & =PenaltyMinutes/4+5*Goals\\ & \;+4*Assists+2*Powerplay\\ & \;Goals+3*GameWinningGoals\\ & \;+1*Plus/Minus+3\\ & \;*ShorthandedGoals \end{array}$$

The formula for forwards was:

$$\begin{array}{rl} FantasyScore & =PenaltyMinutes/4+4*Goals\\ & \;+3*Assists+2*Powerplay\\ & \;Goals+3*GameWinningGoals\\ & \;+1*Plus/Minus+3\\ & \;*ShorthandedGoals \end{array}$$

The formula for goalies was:

$$\begin{array}{rl} FantasyScore & =2*Wins+1*OvertimeLosses\\ & \;+1*Goals+1*Assists\\ & \;+2*Shutouts+Saves/5\\ & \;-1*Losses-.75*GoalsAllowed \end{array}$$

Moreover, the fantasy points for NBA players were not calculated using standard points but based on fantasy league scoring. A standard NBA fantasy league consists of nine categories used for scoring which was utilized in this study. More specifically, the nine-category league fantasy scores were calculated using: (1) points scored by the player, (2) field goal percentage, (3) free throw percentage, (4) three-point field goals, (5) assists, (6) steals, (7) blocks, (8) rebounds, and (9) turnovers.^23^

To track the performance of MLB players, WAR was used instead of fantasy points. WAR values for each MLB player were obtained from FANTASYGRAPHS. WAR is one statistic that assesses a player’s contribution to the team. WAR utilizes various inputs, which that illustrated in the following formula for position players:

$$\begin{array}{rl} WAR & =(BattingRuns+BaseRunningRuns\\ & \;+FieldingRuns+PositionalAdjustment\\ & \;+LeagueAdjustment\\ & \;+ReplacementRuns)/(RunsPerWin) \end{array}$$

Essentially, the formula calls for the sum of batting runs, base running runs, fielding runs, positional adjustment and league adjustment. Replacement runs are added to compare the player’s performance to the replacement level rather than the average player. This sum amounts to runs above replacement (RAR). The RAR is subsequently divided by runs per win to yield WAR. WAR is all-inclusive and provides a useful reference point for comparing two different players or comparing players against themselves from season to season.^24^ Similar to fantasy points, WAR takes multiple statistical measurements to yield one number that reflects a player’s team summary “value”.

The formula for WAR for pitchers was different from positional players. Pitching WAR is based on the number of innings the pitcher threw, park pitched in and fielding independent pitching (FIP). FIP is a measure of a pitcher’s run prevention independent of the performance of their defense. The formula for FIP is illustrated below:

$$\begin{array}{rl} FIP & =((13*HR)+(3*(BB+HBP))\\ & \;-(2*K))/IP+FIPconstant \end{array}$$

Where HR is number of home runs, BB is walks, HBP is hit by pitch, K is strikeouts and IP is innings pitched. The FIP constant is calculated via the following formula:

$$\begin{array}{rl} FIPConstant & =lgERA-(((13*lgHR)\\ & \;+(3*(lgBB+lgHBP))\\ & \;-(2*lgK))/lgIP) \end{array}$$

Where lgERA, is the league earned run average, lgHR is league home runs, lgBB is league’s walks, lgHBP is league hit by pitch, lgK is league strikeouts and lgIP is league innings pitched.^25^

### Interpretation of Findings

For review of data findings, the player’s name, position, date of operation, season played, games played and fantasy score (for NFL, NBA and NHL players) or WAR (for MLB players) were tabulated in a Microsoft Excel 2016 spreadsheet. Next, the average number of games played and fantasy score or WAR prior to and after the date of the index operation were calculated. This enabled the authors to compare a player’s performance against themselves before and after the surgery.

Finally, the average number of games played and the fantasy score or WAR were graphed with respect to each season the player played in as a visual representation of a player’s performance and to discern the pattern of postoperative performance. The average of the number of games played and average fantasy score prior and after surgery were compared to assess how the surgery affected a player’s performance.

Other parameters that were examined included: RTP, career length after the index operation, length of time to return to presurgical level of performance and recovery time. For this study, the RTP was defined as the percentage of athletes that returned to professional play after undergoing the procedure. Career length after the index operation was defined as the number of years played after undergoing surgery.^26^ Recall that the time interval of this study was from 1993 to 2015. Since there were sample players who continued to play after the 2015, their reported career lengths were underestimates.

Length of time to return to presurgical level of performance was defined as the time it took for the player to reach a fantasy value after surgery that was equivalent to his fantasy score the year before surgery. Recovery time was defined as the length of time between surgery and return to competitive play in the patients’ sports.

## RESULTS

**Fig. 1.**
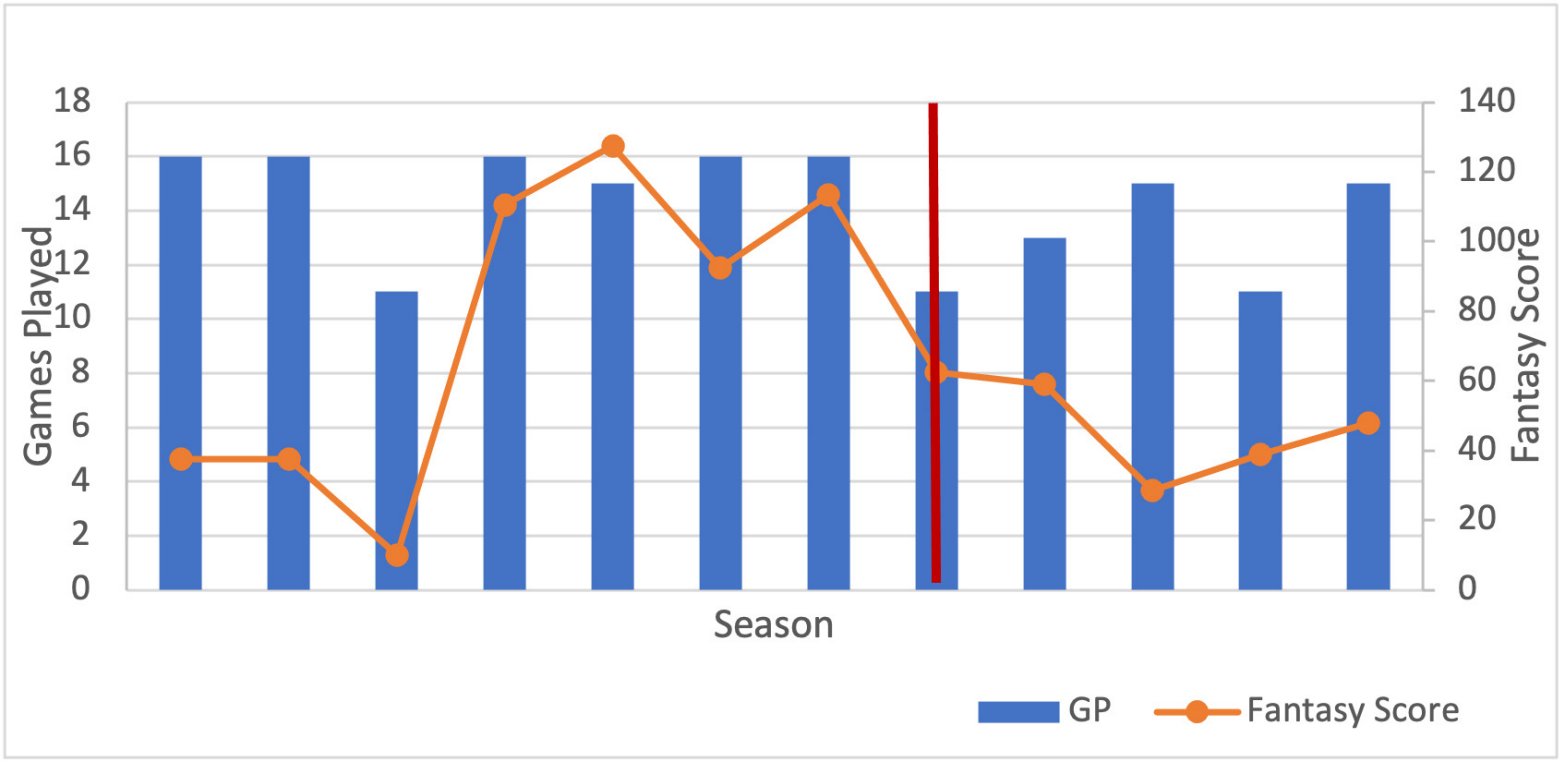
Graph of the number of games played and fantasy score of an example player in the NFL with respect to each season played. Red line marks the year of the index operation.

**Figure 2.1.**
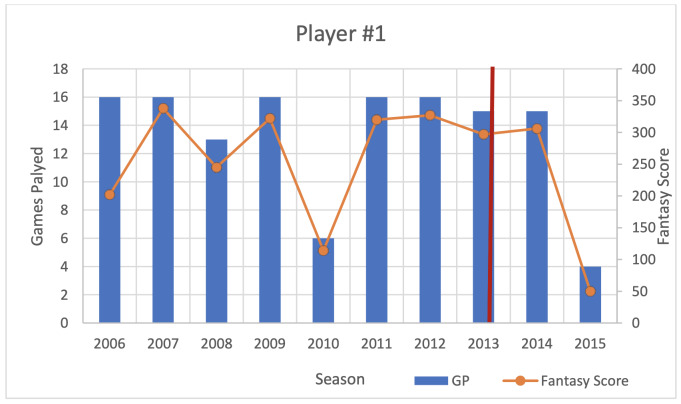
Graph of the number of games played and fantasy score with respect to the season played for player #1.

**Figure 2.4.**
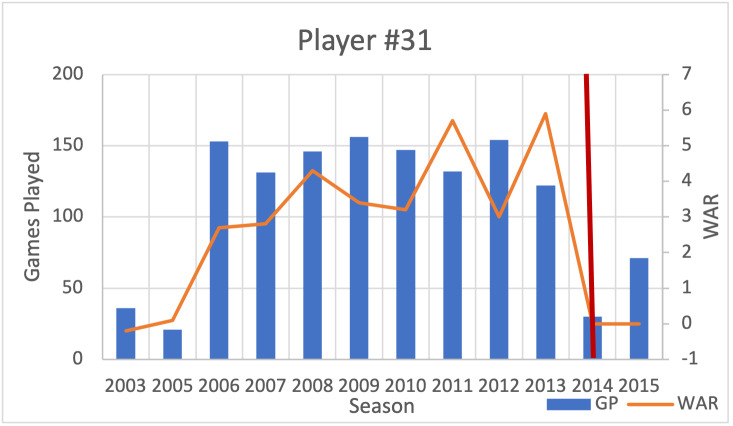
Graph of the number of games played and WAR with respect to the season played for player #31.

A total of 38 (92.7%) professional athlete players met study inclusion criteria of approximately 41 first-identified athletes. Sample players were split into four cohorts (NFL, NBA, NHL and MLB). For each player, the number of games played and fantasy score for each season was tabulated and graphed as illustrated in Table 3 and exemplar Fig. 1, respectively. Note that this player displayed a decrease in the number of games played and fantasy points after the operation. The year of the surgery is marked by a red vertical line. The performance tables and graphs for an example of a professional athlete for each of the major sports analyzed can be viewed in the Appendix (see Appendices A and B with accompanying figures 2.1-2.4).

**Table 3.** Table Outlining the number of games played and fantasy score with respect to each season for example NFL player

Average games played pre-op: 14.63	Average fantasy score pre-op: 73.94
Average games played post-op: 13.5	Average fantasy score post-op: 43.63

The year of the index operation is 2011.

The authors also examined the performance of 14 NFL players. Ten players (71%) exhibited a decrease in the number of games played and fantasy score after undergoing a lumbar discectomy. Next, the authors examined performance outcomes of seven NBA players. Four players (57%) showed a decrease in the number of games played and fantasy score after undergoing the index operation, while one player did not return to play. Notably, there was one (14.3%) NBA player who demonstrated an increase in fantasy value and decrease in the number of games played. In addition, there was one (14.3%) other NBA player who exhibited a decrease in fantasy value, but increased games played.

The authors also examined the statistics of nine NHL players. Four (44.4%) NHL players showed a decrease in the number of games played and fantasy value status post a lumbar discectomy. Two (22.2%) NHL players saw an increase in games played, but they still exhibited decrease in fantasy value. Therefore, six (67%) players were found to have a decrease in fantasy value. Interestingly, three (33%) players exhibited an increase in games played and fantasy value.

Finally, the authors reviewed the statistics of eight MLB players. Seven MLB players (88%) exhibited a decrease in games played and WAR. However, one player (a pitcher) exhibited an increase in games played and a decrease in WAR.

In addition, the performance graphs for all players (available by request from the corresponding author) were generated. In summary, 12 (31%) of all sample players demonstrated an increase in games played and fantasy score or WAR within the first two post-procedure years. However, after the first couple of years a steep decline in performance occurred. Interestingly, this spike in performance after surgery was seen in NFL, NBA and NHL players, but not in MLB players.

Table 4 illustrates a comparison of surgical outcomes between players across the four leagues.

**Table 4.** Comparison of RTP, career length after the index operation, time to return to presurgical level of performance and recovery time between the four leagues

	**NFL**	**NBA**	**NHL**	**MLB**
RTP (%)	64%	86%	89%	88%
Average career length after the index operation (months)	34.8	48	29	44
Recovery time (months)	6	2.8	4.7	12
Average length of time to return to presurgical level of performance (months)	15	12	12	24

## DISCUSSION

Lumbar discectomy and microdiscectomy for LDH herniation are very successful operations in the general population, with clinical success rates ranging from 75 to 90%.^20,23,24^ The outcome of a lumbar discectomy on elite professional players’ performance had not been well studied and many questions exist about how such operation tends to influence professional athletes’ unique demands on their bodies (e.g., high impact jumping, lateral bending, trunk rotation, flexion, extension, rapid acceleration and deceleration forces).^27,28^

A total of 38 players with a confirmed lumbar discectomy or microdiscectomy and missed time were included in our review. The average RTP across the four leagues was 31 of 38 (82%). This was a rate that was consistent with previously published data.^9,10,17,18,29^ Out of the four leagues, a lumbar discectomy or microdiscectomy had the most apparently disabling effect on NFL players because this cohort had the lowest return to play rate (64%) and the second lowest (at 34.8 months) average career length after surgery.

In terms of post-operative performance, NBA players exhibited the longest career length at an average of 48 months, and MLB players had the longest length of time required to return to presurgical level of performance at 24 months. These patterns may be a reflection of the relative physical demands of each sport and differences in medical adherence protocol or differences in medical clearance protocols.

The average recovery time of 2.8 to 12 months after lumbar discectomy in this sample of elite athletes was greater than that in the general population (i.e., typical recommended return to work period between one to four weeks).^30,31^ Such relative differences could be attributed to athletes choosing to undergo surgery in advanced stages of the disease (e.g., when the season ends), which in turn prolongs recovery time. Another reason for a delayed recovery is perhaps the athlete was placed on injury reserve list because a backup was playing their position.

Sample players who returned to play after undergoing a discectomy had lower average games played [14.63 (pre-op) vs 13.5 (post-op)], lower fantasy scores and WAR values [73.94 vs 43.63] compared to prior the operation. This phenomenon could be attributed to athletes not allowing enough time to recover from their operation because there is so much emphasis about “getting back to the game” in addition to the unusual physiologic demands.

Notably, there was one (14.3%) NBA player who demonstrated an increase in fantasy value and decrease in the number of games played as well as one (14.3%) other NBA player who exhibited a decrease in fantasy value, but increased games played. Both of these scenarios appear to be anomalies, although previous studies have shown that physical performance in the NBA tends to decline with age even though postoperative playing time and technical performance remains stable or increased.^32^

The summary performance graphs in the appendix reveals a trend in which some athletes display an increase in performance in the first few years after the operation, followed by a sharp decline. This increase in performance could be attributed to the player now playing pain free. Without pain, the player is better at playing the game. However, our data shows that their performance is expected to decline as time passes.

### Study Limitations

Our study results are subject to several limitations. First, our small sample of male-only professional athletes may not comprise a true representation of all types of elite athletes. Another limitation concerns the inherent variations of reporting sources of performance-based outcomes and complications that may have skewed our outcome measures. Our study lacked age-matched controls which may have provided a more rigorous longitudinal performance representation of aging athletes over time.

A final limitation concerns the use of statistics websites and reliance on publicly accessible news information sources prone to reporting bias. The detailed medical information of each athlete was limited to medical personnel documentation and data regarding some complications or patient specific factors that could have confounded our interpretation of available data was not always available.

## CONCLUSIONS

Based on these study results, the average performance of an elite athlete tends to decrease after undergoing a lumbar discectomy. Although it appears that performance peaks in the initial years after the operation for some players, there is an overall long-term performance decline. More studies are needed to better understand the effects of lumbar discectomy in elite athletes. Future larger-scale prospective studies could also be conducted to define the best treatment and recovery regimens for LDH in different types of elite athletes.

### Conflicts of Interest

None

### Financial Interests

None

## Figures and Tables

**Figure 2.2.**
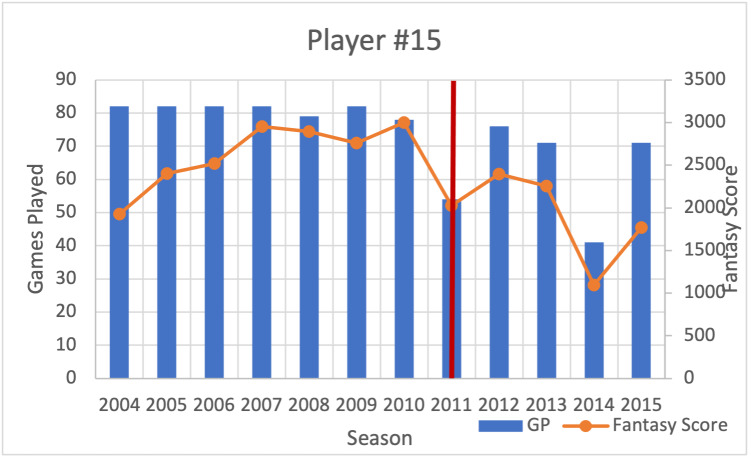
Graph of the number of games played and fantasy score with respect to the season played for player #15.

**Figure 2.3.**
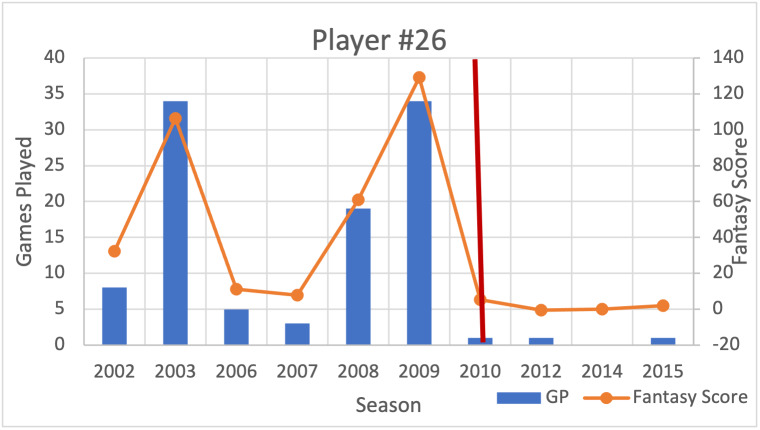
Graph of the number of games played and fantasy score with respect to the season played for player #26.

## Appendix A. Examples of professional athletes (NFL, NBA, NHL and MLB) of average games played and fantasy scores pre- and post-op

### Appendix A.1. Player #1 (NFL)

Player position: Quarterback

Date of operation: December/2013

Average games played pre-op: 14.25

Average fantasy score pre-op: 270.63

Average games played post-op: 9.5

Average fantasy score post-op: 178

### Appendix A.2. Player #15 (NBA)

Player position: Center

Date of operation: April/2011

Average games played pre-op: 81

Average fantasy score pre-op: 2637

Average games played post-op: 62.6

Average fantasy score post-op: 1907

### Appendix A.3. Player #26 (NHL)

Player position: Goaltender

Date of operation: October/2010

Average games played pre-op: 14.86

Average fantasy score pre-op: 50.43

Average games played post-op: 0.67

Average fantasy score post-op: 0.48

### Appendix A.4. Player #31 (MLB)

Player position: outfielder

Date of operation: August/2014

Average games played pre-op: 111.64

Average WAR pre-op: 2.81

Average games played post-op: 71

Average WAR post-op: 0

**Appendix B.** Tables outlining the number of games played and fantasy score with respect to each season<br><br>Appendix B.1. Player #1 (NFL)

SEASON	2006	2007	2008	2009	2010	2011	2012	2013	2014	2015
GP	16	16	13	16	6	16	16	15	15	4
Fantasy Score	202	338	245	322	114	320	327	297	306	50

**Appendix B.2.** Player #15 (NBA)

SEASON	2004	2005	2006	2007	2008	2009	2010	**2011**	2012	2013	2014	2015
GP	82	82	82	82	79	82	78	54	76	71	41	71
Fantasy Score	1927	2402	2519	2953	2897	2758	3003	2024	2395	2255	1094	1767

**Appendix B.3.** Player #26 (NHL)

SEASON	2002	2003	2006	2007	2008	2009	**2010**	2012	2014	2015
GP	8	34	5	3	19	34	1	1	0	1
Fantasy Score	32.25	106.35	11.1	7.95	60.9	129.05	5.4	-0.55	0	2

**Appendix B.4.** Player #31 (MLB)

YEAR	2003	2005	2006	2007	2008	2009	2010	2011	2012	2013	**2014**	2015
GP	36	21	153	131	146	156	147	132	154	122	30	71
WAR	-0.2	0.1	2.7	2.8	4.3	3.4	3.2	5.7	3	5.9	0	0
